# Hyperopic anisometropia with a shorter axial length ipsilateral to the ptotic eye in children with congenital ptosis

**DOI:** 10.1186/s12886-021-02126-8

**Published:** 2021-10-09

**Authors:** Satoshi Ueki, Yuji Suzuki, Megumi Kiyokawa, Takako Hanyu, Takeo Fukuchi

**Affiliations:** 1 Division of Ophthalmology and Visual Science, Graduate School of Medical and Dental Sciences, Niigata University, Asahimachi-dori 1-757, Chuo-ku, Niigata, 951-8510 Japan; 2 Center for Integrated Human Brain Science, Brain Research Institute, Niigata University, Asahimachi-dori 1-757, Chuo-ku, Niigata, 951-8585 Japan; 3 Hanyu Clinic, Igarashi-higashi 1-1-15, Nishi-ku, Niigata, 950-2045 Japan

**Keywords:** Congenital ptosis, Hyperopic anisometropia, Shorter axial length

## Abstract

**Background:**

To investigate the clinical characteristics of children with congenital ptosis, with particular attention given to the incidence of anisometropia, and the difference in axial length (AL) between the right and left eyes.

**Methods:**

The medical charts of 55 patients with congenital ptosis at Niigata University Medical and Dental Hospital were retrospectively analyzed. Clinical characteristics, including age, cycloplegic refraction, AL, and the presence of amblyopia and its causes were analyzed.

**Results:**

Age at the initial visit was 16 ± 20 (mean ± standard deviation, the same applies below) months. Of the 49 patients whose cycloplegic refraction was measured, hyperopic anisometropia, defined as ≥ one-diopter difference in spherical equivalent (SE), was observed in 1/11, 9/27 and 5/11 patients with bilateral, right, and left ptosis, respectively. Among 14/38 patients with hyperopic anisometropia involving unilateral ptosis, 13 demonstrated a larger SE in the ptotic eye than in the non-ptotic eye. The inter-eye difference in AL (AL of the ptotic eye minus that of the non-ptotic eye) in six patients with unilateral ptosis and hyperopic anisometropia ipsilateral to the ptotic eye (-0.29 ± 0.40 mm) was significantly smaller than that in three patients with unilateral ptosis and no hyperopic anisometropia (0.38 ± 0.29 mm).

**Conclusions:**

At our institute, children with congenital ptosis had a high incidence of hyperopic anisometropia ipsilateral to the ptotic eye. Furthermore, this condition was associated with a shorter axial length. These results indicate that refractive correction for hyperopic anisometropia is important for proper visual development in children with congenital ptosis.

## Background

Congenital ptosis is a bilateral or unilateral eyelid disorder characterized by drooping of the upper eyelid from birth that can cause functional and cosmetic disabilities. Ophthalmologic manifestations associated with congenital ptosis are amblyopia, refractive error, and strabismus. Children with congenital ptosis may develop amblyopia induced by refractive error, as well as ptosis itself and strabismus. A previous study showed that about 50% of patients with congenital ptosis and amblyopia had only refractive error [1], and therefore assessment of refractive error is crucial in clinical practice for children with congenital ptosis. However, the type of refractive error associated with congenital ptosis has differed in previous studies [2–5]. Anisometropia, defined as an inter-eye difference in refractive error, is likely to develop and may cause anisometropic amblyopia especially in patients with unilateral ptosis. However, much attention has not been paid to anisometropia associated with congenital ptosis, and, furthermore, it remains unknown how ptosis affects eye growth and induces refractive error. In this study, we investigated cycloplegic refraction to focus on anisometropia, and axial length to reveal an association between eyeball shape and refractive error induced by ptosis.

## Methods

We retrospectively reviewed the charts of 66 children under 15 years old who were diagnosed with congenital ptosis at Niigata University Medical and Dental Hospital from January 2005 through December 2017. Each patient’s medical history was obtained at the initial visit. Ophthalmic evaluations, including visual acuity using Teller acuity cards or Landolt ring (if possible), slit-lamp examination, and fundus ophthalmoscopy with pupil dilatation, were also performed at the initial visit. Congenital ptosis was diagnosed by the presence of unilateral or bilateral drooping of the upper eyelid from birth and no limitation of ocular motility. Exclusion criteria were congenital oculomotor nerve palsy, congenital fibrosis of the extraocular muscles, and Marcus-Gun phenomenon. Among 66 patients diagnosed with congenital ptosis, 11 patients with neurological disease or whose past medical history was unknown were excluded. Informed consent was obtained using the opt-out method. The study was approved by the Institutional Review Board/Ethics Committee at Niigata University (Registration No. 2020-0270) and followed the tenets of the Declaration of Helsinki. We recorded clinical characteristics, including age, sex, laterality, cycloplegic refraction (if performed), axial length (AL) (if performed; assessed with an IOLMaster 500^TM^ (Carl Zeiss Meditec AG, Jena, Germany)), the presence of amblyopia and its cause, treatments, and follow-up period (the latest follow-up was in December 2019). Anisometropia was defined as proposed by Chia et al. [6] Hyperopic anisometropia was defined as ≥ one-diopter (D) difference in spherical equivalent (SE). Myopic anisometropia was defined as ≥ 3 D difference in SE. Astigmatic anisometropia was defined as ≥ 1.5 D difference in cylinder. Unilateral amblyopia was defined as ≥ 2-line difference in best-corrected visual acuity using Landolt ring during follow-up period.

In patients with unilateral ptosis, the paired *t*-tests were performed between SE of the eye with or without ptosis, and cylinder of the eye with or without ptosis. In patients who underwent AL measurement, Pearson’s correlation and the unpaired *t*-test were performed to analyze the correlation between differences in SE and in AL, and to compare AL between patients with or without anisometropia. These analyses were conducted using SigmaPlot 14 (Systat Software, San Jose, CA, USA). A p-value < .05 was considered statistically significant.

## Results

The age of the 55 study patients at the initial visit was 16 ± 20 months (mean ± standard deviation (SD), the same applies below). There were 40 males and 15 females. Ptosis was bilateral in 12 patients, right-sided in 30, and left-sided in 13. Cycloplegic refraction was measured in 49 patients (bilateral in 11 patients, right-sided in 27, and left-sided in 11) (the mean ± SD and median ages at the time of measurement were 32 ± 19 and 31 months, respectively); the SE of the eye with ptosis was 1.71 ±1.55 D, the SE of the eye without ptosis was 1.35 ± 1.20 D, the cylinder of the eye with ptosis was -1.31 ± 0.79 D, and the cylinder of the eye without ptosis was -1.07 ± 0.96 D. In 38 patients with unilateral ptosis, the SE of the eye with ptosis was 2.02 ±1.33 D, and the cylinder of the eye with ptosis was -1.33 ± 0.85 D (Table 1). SE of the ptotic eye was significantly larger than that of the non-ptotic eye in patients with unilateral ptosis (*P* = .00, paired *t*-test), whereas cylinder of the ptotic eye was not significantly different from that of the non-ptotic eye in patients with unilateral ptosis (*P* = .08, paired *t*-test) (Table 1). Hyperopic anisometropia was observed in 1/11, 9/27, and 5/11 patients with bilateral, right, and left ptosis, respectively. Among 14/38 patients with hyperopic anisometropia involving unilateral ptosis, 13 demonstrated a larger SE in the ptotic eye than in the non-ptotic eye. Myopic anisometropia was not observed in any of the 49 patients. Astigmatic anisometropia was observed in 1/11, 2/27, and 2/11 patients with bilateral, right, and left ptosis, respectively. Among 4/38 patients with astigmatic anisometropia patients involving unilateral ptosis, three had a larger cylinder in the ptotic eye than in the non-ptotic eye. In one patient with left ptosis, the cylinder of the right eye was -0.25 D and that of the left eye was -2.25 D. In one patient with right ptosis, the cylinder of the right eye was -2.0 D and that of the left eye was -0.5 D. In another patient with right ptosis, the cylinder of the right eye was -2.75 D and that of the left eye was -0.5 D. The patient with left ptosis had amblyopia on account of an anisometropia in both the hyperopic SE and astigmatism.

**Table 1:** Refractive errors of patients with unilateral congenital ptosis who underwent cycloplegic refraction measurement

	Ptotic eye (*n* = 38)	Non-ptotic eye (*n* = 38)	Statistical significance (*P* < .05)
SE (diopters)	2.02 ± 1.33	1.35 ± 1.20	*P* = .00
Cylinder (diopters)	-1.34 ± 0.85	-1.07 ± 0.96	NS (*P* = .08)

The data are presented as mean ± SD

*NS* not significant

Among 14 patients with unilateral ptosis and hyperopic anisometropia, eight patients had mild ptosis not covering the pupil and six patients had moderate ptosis covering the part of pupil. No patients had severe ptosis covering the whole of pupil. In follow-up analysis of cycloplegic refraction, four of 14 patients with unilateral ptosis and hyperopic anisometropia continued to experience hyperopic anisometropia whereas seven patients demonstrated resolution of hyperopic anisometropia throughout the follow-up period. (the mean ± SD and median follow-up durations were 91 ± 54 and 98 months, respectively). Residual three patients did not undergo repeated cycloplegic refraction measurements throughout the follow-up period. Nine had started wearing glasses after diagnosis of hyperopic anisometropia. Five underwent surgery for ptosis; hyperopic anisometropia resolved before the surgery in three patients and hyperopic anisometropia persisted after the surgery in one patient. In one patient with resolution of hyperopic anisometropia, astigmatic anisometropia developed during follow-up period. In another patient with resolution of hyperopic anisometropia, coexisting astigmatic anisometropia persisted during follow-up.

In patients with unilateral ptosis, given unilateral amblyopia defined as ≥ 2-line difference in best-corrected visual acuity during follow-up period, six patients had hyperopic anisometropic amblyopia, one had astigmatic anisometropic amblyopia, four had ametropic amblyopia, three had deprivation amblyopia due to ptosis, and one had strabismic amblyopia (Table 2). In three of four patients with ametropic amblyopia, the ptotic eyes had greater hyperopia whereas < 1 D difference in SE. Residual one patient with ametropic amblyopia had hyperopia, and < 20/30 in best-corrected visual acuity in both eyes.

**Table 2:** Prevalence of unilateral amblyopia in patients with unilateral ptosis

	Number of patients
Hyperopic anisometropic amblyopia	6
Astigmatic anisometropic amblyopia	1
Ametropic amblyopia	4
Deprivation amblyopia due to ptosis	3
Strabismic amblyopia	1

Unilateral amblyopia was defined as ≥ 2-line difference in best-corrected visual acuity using Landolt ring during follow-up period

**Fig. 1:**
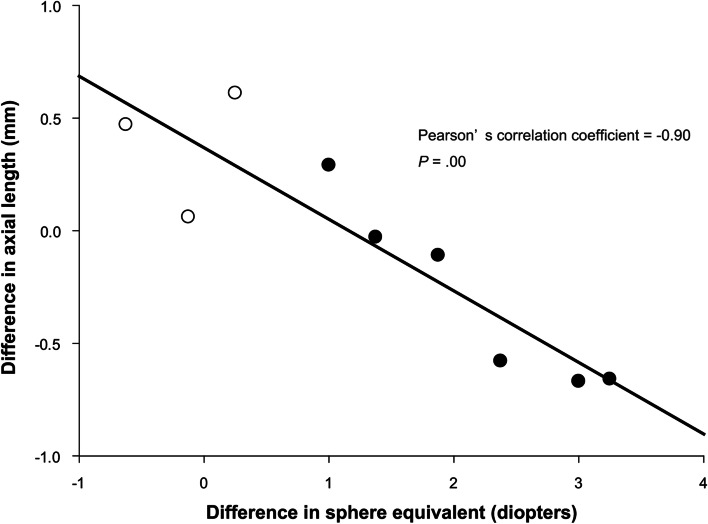
Differences in AL and in SE between the ptotic and the non-ptotic eyes. For both parameters, differences were calculated as the value of the ptotic eye minus the value of the non-ptotic eye. Filled circles represent the values of patients with ≥ 1 D difference in SE. Open circles represent the values of patients with < 1 D difference in SE. The Pearson’s correlation coefficient is -0.90 (*P* = .00). The linear regression line is shown

AL measurement with an IOLMaster 500^TM^ (Carl Zeiss Meditec AG, Jena, Germany) was performed in six patients with unilateral ptosis and hyperopic anisometropia ipsilateral to the ptotic eye, and in three patients with unilateral ptosis without hyperopic anisometropia. The mean ± SD and median ages at the time of measurement of AL were 94 ± 24 and 101 months, respectively. The analysis of the Pearson’s correlation showed a negative correlation between the difference in SE with cycloplegia (SE of the ptotic eye minus that of the non-ptotic eye) against the difference in AL (AL of the ptotic eye minus that of the non-ptotic eye). The Pearson’s correlation coefficient was -0.90 (*P* = .00) (Fig. 1). The difference in AL in patients with unilateral ptosis with hyperopic anisometropia ipsilateral to the ptotic eye was significantly smaller than that in patients with unilateral ptosis and no hyperopic anisometropia (*P* = .04, unpaired *t*-test), whereas the ALs of the ptotic and non-ptotic eyes were not significantly different between the two groups, respectively (*P* = .12 and *P* = .41, respectively, unpaired *t*-test) (Table 3). The mean ± SD and median durations between the times of measurement of AL and cycloplegic refraction were 10 ± 12 and 8 months.

**Table 3:** Differences in AL between unilateral ptosis patients with ≥ 1 D vs. < 1 D difference in SE

AL (mm)	≥ 1 D (*n* = 6)	< 1 D (*n* = 3)	Statistical significance (*P* < .05)
Ptotic eye	22.46 ± 0.37	23.65 ± 1.68	NS (*P* = .12)
Non-ptotic eye	22.75 ± 0.40	23.27 ± 1.41	NS (*P* = .41)
Difference (AL of the ptotic eye minus that of the non-ptotic eye)	-0.29 ± 0.40	0.38 ± 0.29	*P* = .04

The data are presented as mean ± SD

*NS* not significant

## Discussion

Our study revealed a high incidence of comorbidity between hyperopic anisometropia and hyperopic anisometropic amblyopia ipsilateral to the ptotic eye. Furthermore, in patients with unilateral ptosis, SE of the ptotic eyes was significantly larger than that of the non-ptotic eyes. These results indicate that refractive correction that takes hyperopic anisometropia into account is important for managing visual development in children with congenital ptosis. Our findings are consistent with those of a recent report on refractive error in congenital ptosis, which found a high incidence of anisometropia; however, that study mainly focused on the prevalence of myopic anisometropia [2]. A recent meta-analysis of patients with congenital ptosis showed that among various types of refractive error, myopia was the most prevalent (30.2%), followed by anisometropia (17.3%) [7]. Differences among reports in the prevalence of refractive errors may be due to variations in patient age at the time of cycloplegic refraction measurement. In this study, cycloplegic refraction was assessed at a median age of 31 months. Huo et al assessed cycloplegic refraction at an average age of 16.83 years and reported a high incidence of myopia in children with congenital ptosis [3]. In our study, four patients with hyperopic anisometropia ipsilateral to the ptotic eye demonstrated persistence of the condition for the entire follow-up period (median duration, 98 months). On the other hand, the aforementioned meta-analysis showed that 22.2% of children with congenital ptosis had astigmatism [7]. Although astigmatism was also frequently observed in other studies focusing on the clinical characteristics of congenital ptosis [4, 5], our study showed a low prevalence of astigmatic anisometropia.

Furthermore, this study revealed that hyperopic anisometropia ipsilateral to the ptotic eye was associated with a shorter AL although analysis of unpaired *t*-test had small numbers. Emmetropization is the postnatal growth of the eye toward emmetropia, and further eye growth results in the progression of myopia. Recent investigations in a monkey model of myopia revealed that myopia progression was induced by peripheral hyperopic defocus [8, 9]. The eyelid narrowing in ptosis induces a pinhole effect, and we hypothesize that this effect increases depth of focus and thereby reduces hyperopic defocus attributable to myopia progression in ptosis patients. Our patients with unilateral ptosis and hyperopic anisometropia did not have severe ptosis covering the whole of pupil.

In our investigation, ptosis affected the right side more often than the left. By contrast, Griepentrog et al reported that left ptosis was much more common [10]. The small number of patients in our study might have contributed to the discrepancy in these results.

This study has several limitations. First, it was conducted at a single institute and included only 55 patients. We are planning to perform further studies to collect additional patients. Second, the AL measurement performed in this study consisted only of a one-dimensional assessment of eyeball shape. Further research that includes a three-dimensional analysis is necessary to reveal detailed changes in eyeball shape in patients with hyperopic anisometropia ipsilateral to the ptotic eye.

## Conclusions

We revealed that children with congenital ptosis at our institute had a high incidence of hyperopic anisometropia ipsilateral to the ptotic eye. Furthermore, of 14 patients with unilateral ptosis and hyperopic anisometropia, four continued to have hyperopic anisometropia throughout the follow-up period. In addition, we revealed that hyperopic anisometropia ipsilateral to the ptotic eye was associated with a shorter AL. These results indicate that refractive correction of hyperopic anisometropia is important for proper visual development in children with congenital ptosis.

## Data Availability

Authors can confirm that all relevant data are included int the article. The data that support the findings of this study are available on request from the corresponding author.

## Abbreviations

AL: Axial length
SE: Spherical equivalent
D: Diopter
SD: Standard deviation
